# Search for Potentially Virulent *Staphylococcus epidermidis* Isolated from Healthy Human Skin

**DOI:** 10.3390/ijms27156611

**Published:** 2026-07-24

**Authors:** Marco Antonio Hernández-Valenzuela, Ana Luisa Cruz-Morales, Alma Nelly Diaz-Herreros, Alejandro A. Hidalgo, Mario Eugenio Cancino-Diaz, Juan Carlos Cancino-Diaz

**Affiliations:** 1 Laboratorio de Inmunomicrobiología, Departamento de Microbiología, Escuela Nacional de Ciencias Biológicas del Instituto Politécnico Nacional, Mexico City 11340, Mexico; mhernandezv2212@alumno.ipn.mx (M.A.H.-V.); acruzm1405@alumno.ipn.mx (A.L.C.-M.); 2 Laboratorio de Inmunología Aplicada, Departamento de Inmunología, Escuela Nacional de Ciencias Biológicas del Instituto Politécnico Nacional, Mexico City 11340, Mexico; ndiazh1917@alumno.ipn.mx (A.N.D.-H.); mcancinod@ipn.com (M.E.C.-D.); 3 Escuela de Química y Farmacia, Facultad de Medicina, Universidad Andres Bello, Santiago 8370134, Chile; alejandro.hidalgo@unab.cl

**Keywords:** *Staphylococcus epidermidis*, biofilm, virulent-like, catheter, skin, healthy individuals

## Abstract

Genomic studies suggest that the skin microbiota of healthy individuals may harbor *Staphylococcus epidermidis* strains with putative virulence-associated characteristics, and that contamination of medical devices can originate from the patient’s own skin microbiota. However, the virulence potential of these strains and their ability to contaminate medical devices have not been directly validated experimentally. Therefore, this study evaluated whether *S. epidermidis* isolates recovered from the skin of healthy individuals possess the capacity to colonize implanted catheters and form device-associated biofilms. Fifty-two isolates obtained from five dermatologically healthy individuals were phenotypically and genotypically characterized. Principal component analysis (PCA) was used to visualize the distribution of isolates according to their phenotypic and genotypic characteristics. Based on biofilm-forming capacity and antimicrobial resistance/susceptibility profiles, the isolates were assigned to four categories: virulent-like (*n* = 3), low virulent-like (*n* = 7), commensal-like (*n* = 20), and non-virulent-like (*n* = 22). Representative isolates from the resulting groups were evaluated using a catheter contamination assay in a mouse back model (CCAMM). In this model, virulent-like isolates exhibited levels of catheter colonization and device-associated biofilm formation comparable to those observed for the reference strain RP62A, whereas the non-virulent-like isolate displayed substantially lower biofilm formation. In conclusion, these findings suggest that the skin of dermatologically healthy individuals may harbor *S. epidermidis* isolates with virulence-associated traits and highlight its potential role as a reservoir of isolates capable of contaminating implanted medical devices.

## 1. Introduction

*Staphylococcus epidermidis* is a Gram-positive bacterium that colonizes the skin and mucosal of mammals. In humans, colonization begins shortly after birth [1,2]. As a member of the skin microbiota, *S. epidermidis* coexists and interacts with numerous microbial species, contributing to skin homeostasis and protecting against colonization by pathogenic microorganisms [3,4]. For example, *S. epidermidis* can inhibit the growth of pathogenic bacteria such as *Staphylococcus aureus* and prevent the overgrowth of *Cutibacterium acnes*, which has been associated with the development of acne vulgaris [5,6]. *S. epidermidis* interacts with skin cells (mainly keratinocytes) and stimulates the production of antimicrobial peptides [3]. These peptides kill bacteria that are foreign to the microbiota, aiding in the prevention of skin infections. In addition, *S. epidermidis* also contributes to the training of the skin’s immune system by maintaining immune cells in an activated state against foreign agents [7]. In inflammatory skin diseases, such as psoriasis and atopic dermatitis, it has been suggested that *S. epidermidis* plays a role in controlling inflammation [7].

In recent years, however, *S. epidermidis* has been isolated more frequently from hospitalized patients, causing local or systemic infections [4,8,9]. Hospitalized neonates are particularly susceptible to these infections due to their immature immune systems [10]. In such cases, infection is commonly associated with the contamination of medical devices, including needles, catheters, and prosthetic heart valves [11,12,13]. The ability of *S. epidermidis* to adhere to plastic and polymer surfaces together with its ubiquitous presence on human skin facilitates the contamination of medical devices and the subsequent formation of biofilms [14,15]. Biofilm formation protects *S. epidermidis* from both antimicrobial agents and host immune defenses by creating a structured extracellular matrix that limits antibiotic penetration, promotes bacterial persistence, and impairs the ability of immune cells to eliminate the bacteria [16,17]. As a result, these biofilms are difficult to eradicate, and antibiotic treatment is often unsuccessful. *S. epidermidis* has therefore become a significant health concern, leading to prolonged hospital stays and increased costs for public health systems [18]. The exopolysaccharide PIA/PNAG (Polysaccharide Intercellular Adhesin/Poly-β-1,6-N-acetylglucosamine) is a major component of the *S. epidermidis* biofilm matrix. Its synthesis is mediated by the icaADBC operon, in which the IcaA and IcaD proteins play essential roles in the biosynthesis of this exopolysaccharide [19]. Biofilm formation has been strongly associated with clinical *S. epidermidis* isolates, particularly those involved in device-associated infections, whereas most commensal isolates recovered from healthy skin are non-biofilm producers under standard in vitro conditions [18]. This supports the importance of biofilm formation as a relevant virulence-associated trait in this species. However, PIA/PNAG production is not the only mechanism involved in *S. epidermidis* biofilm development. Recent studies have shown that *S. epidermidis* can also form biofilms through PIA/PNAG-independent mechanisms, particularly in *ica*A-negative isolates. In these strains, exposure to exogenous proteases can induce protein-dependent biofilm formation, in which proteinaceous components such as the accumulation-associated protein (Aap) contribute to intercellular adhesion and biofilm accumulation after proteolytic processing [20,21]. In this context, trypsin has been used as an experimental proteolytic stimulus to assess whether exposure to an exogenous protease can induce or enhance Aap-mediated biofilm formation in commensal *S. epidermidis* isolates that do not produce biofilm under standard in vitro conditions.

*S. epidermidis* lacks specialized mechanisms to efficiently breach intact skin barriers. Consequently, contamination of medical devices represents a critical step in the development of *S. epidermidis* infections in hospitalized patients [22]. Despite the clinical importance of these device-associated infections, the origin of the strains responsible for medical device contamination remains incompletely understood [23]. Due to its high prevalence on human skin, it has been suggested that *S. epidermidis* infections may originate either from the skin microbiota of hospitalized patients or from healthcare workers [24]. Several studies have focused on determining the source of *S. epidermidis* strains involved in medical device contamination through comparative analyses of isolates recovered from infected patients and those obtained from the skin of dermatologically healthy individuals [25]. These studies indicate that isolates from infected patients generally exhibit greater biofilm-forming capacity and higher antibiotic resistance compared with isolates obtained from the skin of dermatologically healthy individuals, suggesting that these characteristics may contribute to the differentiation of infection-associated and commensal isolates and may represent important virulence-associated traits of *S. epidermidis* [26,27,28,29]. Many of these studies are based on the analysis of a single isolate from each patient or healthy subject, which limits their ability to determine the origin of medical device contamination by this bacterium.

In this context, a study by Zhou et al. [30] focused on isolating *S. epidermidis* from different anatomical skin sites of five healthy individuals in order to understand the structure and dynamics of *S. epidermidis* populations through genomic sequencing. They showed that each individual harbored a unique *S. epidermidis* population composed of multiple founders or clones; these coexisted across different anatomical sites and exhibited high genetic diversity, including a mixture of commensal and putative antibiotic-resistant bacteria [30]. This earlier study supports the hypothesis that the skin of healthy individuals may harbor *S. epidermidis* strains with virulence-associated characteristics, mixed with commensal strains. Such strains with putative virulence-associated characteristics may contribute to the contamination of medical devices and the subsequent development of infection. However, this hypothesis has not yet been directly experimentally tested. Given the widespread distribution of *S*. *epidermidis* across multiple anatomical sites in healthy, non-hospitalized individuals, it remains unclear whether isolates exhibiting virulence-associated phenotypes, such as biofilm formation and antibiotic resistance, are more likely to contaminate medical devices compared with commensal strains [22]. Therefore, the aim of this study was to determine whether *S. epidermidis* isolates recovered from the skin of healthy individuals and characterized by enhanced biofilm-forming ability and antibiotic resistance exhibit an increased capacity to colonize and contaminate medical devices. To address this question, phenotypic and genotypic characterization, multivariate analysis, and an in vivo catheter contamination model were employed to identify isolates exhibiting virulence-associated traits and to evaluate their ability to form device-associated biofilms.

## 2. Results

### 2.1. Distribution of Staphylococcus epidermidis Isolates

**Figure 1:**
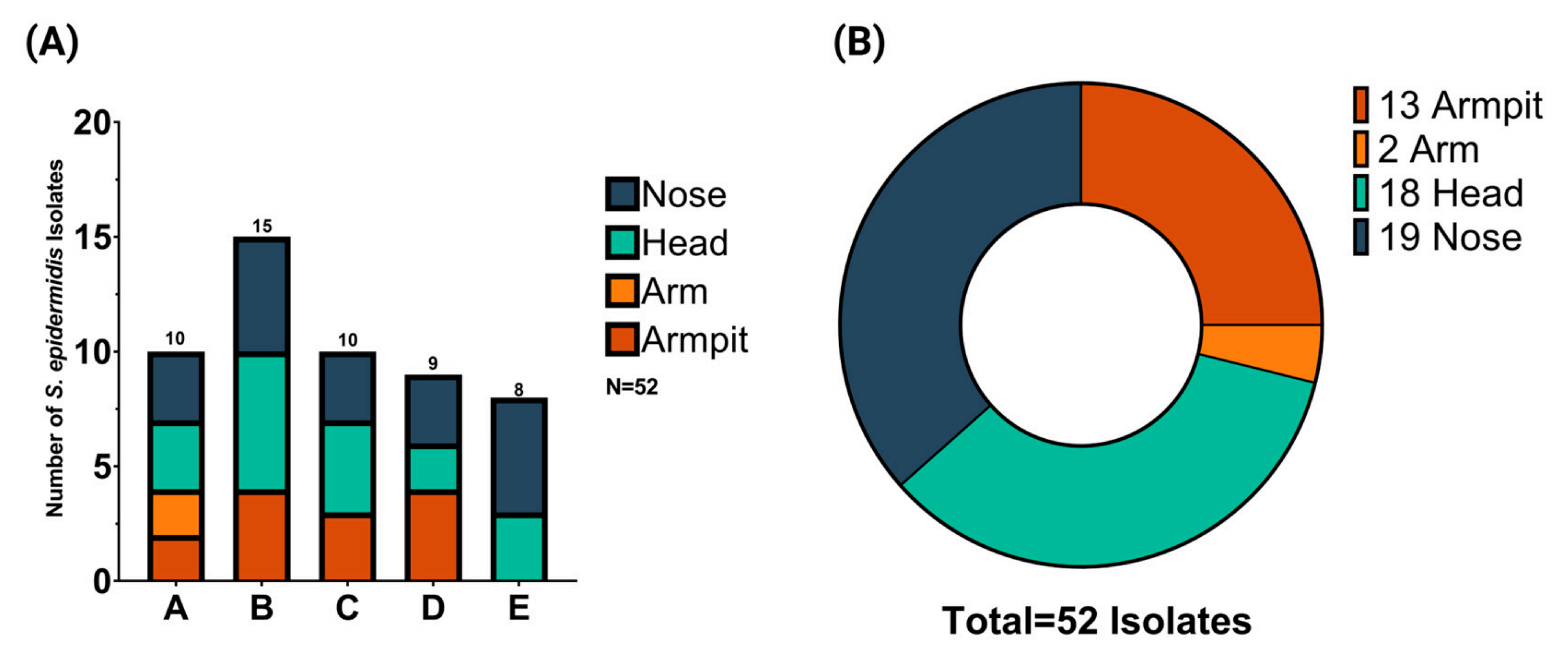
Distribution of *Staphylococcus epidermidis* isolates obtained from different anatomical sites in healthy individuals. (**A**) Number of isolates recovered from each individual (A–E) and their distribution across anatomical sites (nose, head, arm, and armpit). (**B**) Overall proportion of isolates according to anatomical site. A total of 52 isolates were analyzed.

*S. epidermidis* isolates were collected from the skin of the head, arms, nose, and armpit of five dermatologically healthy individuals (Figure 1A). Subject B yielded the highest number of isolates compared to the other subjects. In total, 52 *S. epidermidis* isolates were collected. The head and nasal areas yielded the most isolates, while the axillary region had the fewest (Figure 1B).

### 2.2. Phenotypic Characterization of Staphylococcus epidermidis Isolates

**Figure 2:**
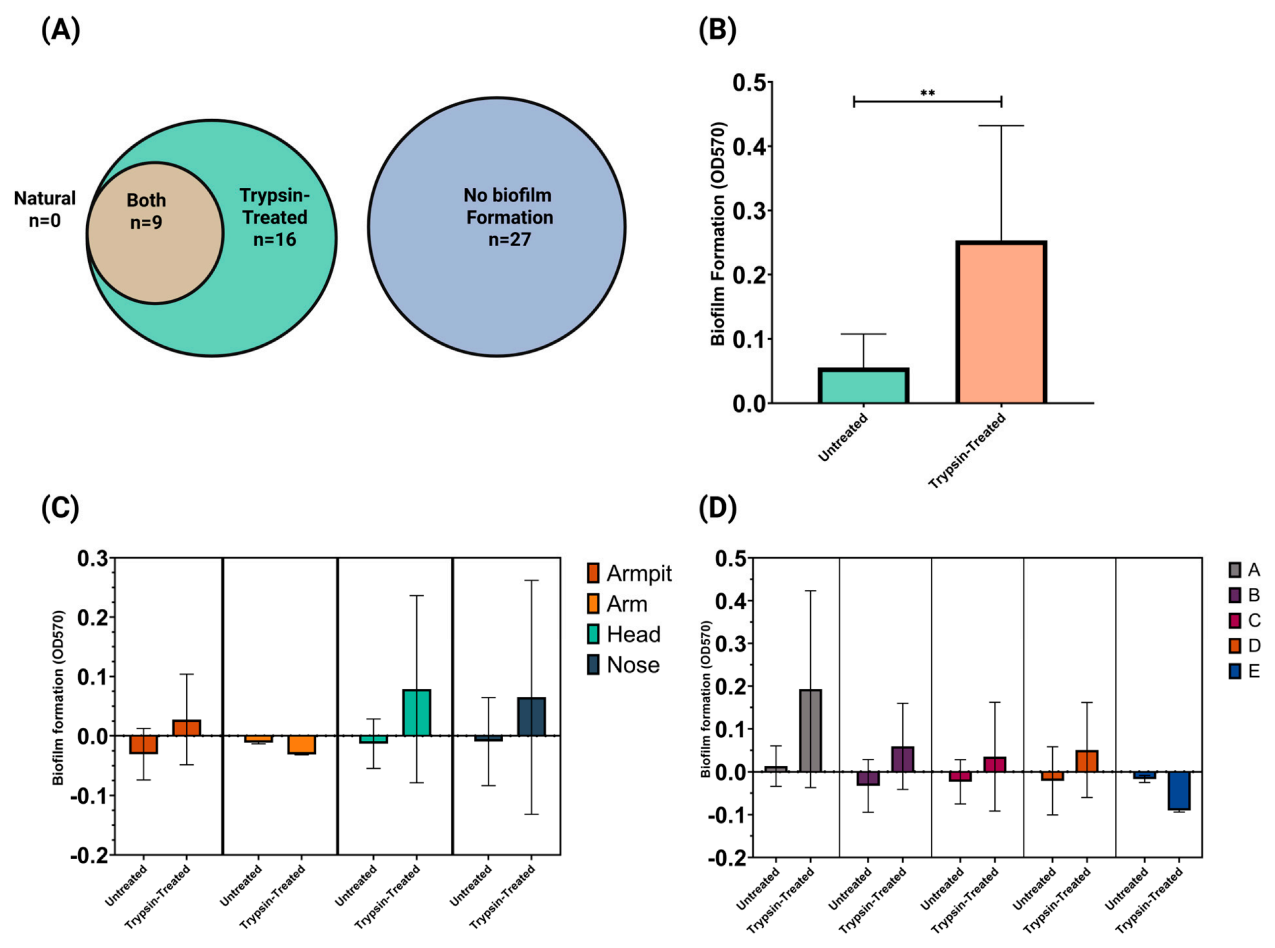
Biofilm formation in *Staphylococcus epidermidis* isolates. (**A**) Distribution of biofilm-forming capacity among the isolates (*n* = 52 isolates). (**B**) Comparative analysis of biofilm production in isolates able to form biofilm under both natural and trypsin-treated conditions (*n* = 9 isolates). Bars represent mean ± SEM. Statistical significance was determined using a paired Student’s *t*-test (** *p* < 0.01). (**C**) Descriptive comparison of biofilm formation under natural and trypsin-treated conditions according to anatomical origin, including armpit (*n* = 13 isolates), arm (*n* = 2 isolates), head (*n* = 18 isolates), and nose (*n* = 19 isolates). (**D**) Descriptive comparison of biofilm formation under natural and trypsin-treated conditions according to subject, including subjects A (*n* = 10 isolates), B (*n* = 15 isolates), C (*n* = 10 isolates), D (*n* = 9 isolates), and E (*n* = 8 isolates).

In vitro biofilm formation was evaluated under natural and trypsin-treated conditions. Among the 52 *S. epidermidis* isolates analyzed, 27 isolates (51.9%) did not form biofilm under either condition (Figure 2A). In contrast, 9 isolates (17.3%) formed weak biofilm under natural conditions, whereas 16 isolates (30.7%) formed biofilm only after trypsin treatment. Notably, the 9 isolates that produced weak biofilm under natural conditions shifted to moderate biofilm production after trypsin treatment (Figure 2B). Comparison of biofilm formation among anatomical sites (Figure 2C) and subjects (Figure 2D) did not reveal a clear pattern suggesting that isolates from any particular anatomical site or individual exhibited greater biofilm formation under natural conditions.

### 2.3. Genotypic Characterization of Staphylococcus epidermidis Isolates

The distribution of the genes *ica*A, *ica*D, *aap*, IS256, *mec*A, and *sar*A among the isolates did not reveal any clear clustering pattern. Nevertheless, *ica*A, *ica*D, and *aap* genes were detected in the majority of isolates, whereas *mec*A, IS256, and *sar*A were detected only in a minority of isolates (Table S1).

### 2.4. Search for Virulent-like Isolates Based on Phenotypic and Genotypic Data

**Figure 3:**
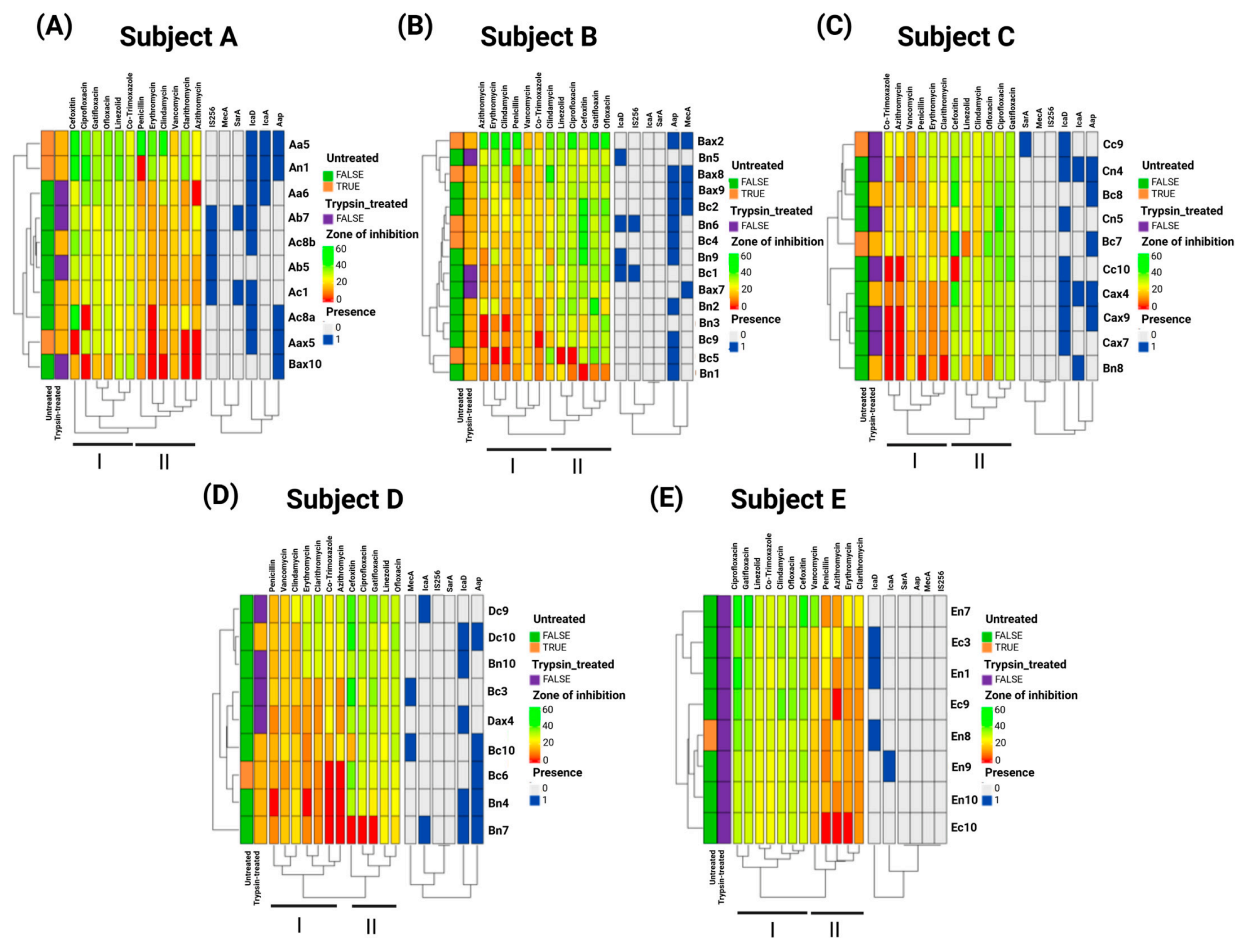
Subject-specific hierarchical clustering of *S. epidermidis* isolates. Heatmap showing antibiotic susceptibility profiles, biofilm formation capacity (natural and trypsin-treated), and presence of virulence-associated genes. Each row represents one bacterial isolate. (**A**) Subject A (*n* = 10 isolates), (**B**) Subject B (*n* = 15 isolates), (**C**) Subject C (*n* = 10 isolates), (**D**) Subject D (*n* = 9 isolates), and (**E**) Subject E (*n* = 8 isolates).

To further explore the relationships between isolates, hierarchical clustering analysis was performed using phenotypic and genotypic data. When combining these datasets for each healthy subject, a heterogeneous distribution of isolates was observed across individuals, with no defined clustering pattern; this suggests subject-specific profiles not shared between individuals (Figure 3A–E). Within each subject, two main groups (I and II) were identified based on antibiotic resistance/susceptibility profiles, indicating that each subject exhibited a unique resistance profile, with variability in antibiotic susceptibility observed between individuals. When analyzed by anatomical region across all subjects, a similar pattern was observed, with a heterogeneous distribution of isolates. Again, this suggests clear segregation of isolates into two groups based on antibiotic resistance/susceptibility, and a wide distribution across subjects (Figure S1). However, it is important to note that, although a heterogeneous distribution of isolates was observed among subjects and anatomical sites, some isolates may belong to the same clonal lineage, since the 52 isolates were not subjected to deeper genomic typing in this study. Therefore, the actual number of independent isolates and the extent of their distribution may be lower than that inferred from the isolate-level analysis.

**Figure 4:**
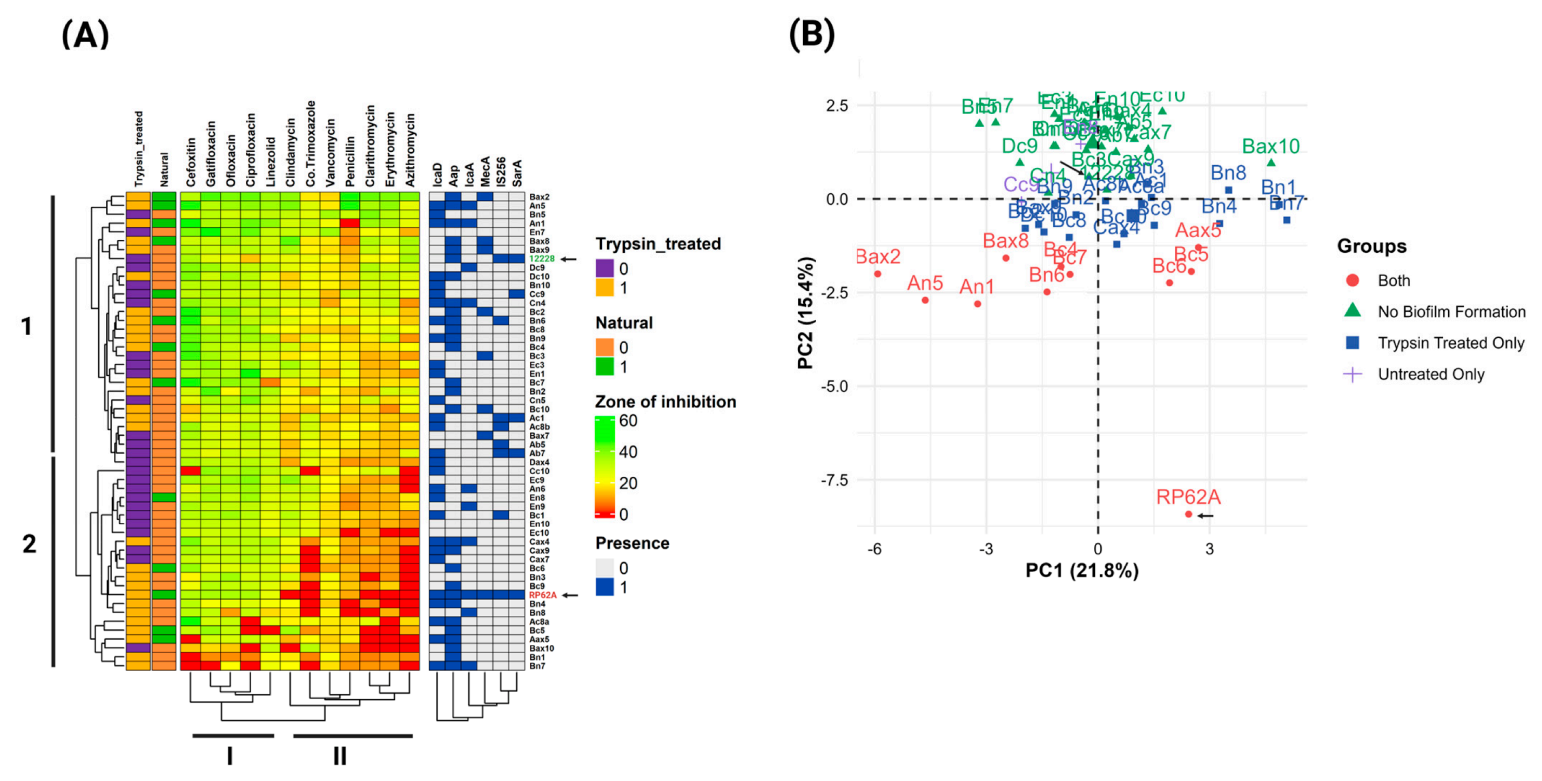
Multivariate analysis of phenotypic and genotypic characteristics of *S. epidermidis* isolates. (**A**) Hierarchical clustering heatmap showing antibiotic susceptibility profiles, biofilm formation capacity (natural and trypsin-treated), and presence of virulence-associated genes in the 52 isolates analyzed, together with the reference strains RP62A and ATCC12228. (**B**) Principal component analysis (PCA) illustrating the distribution of isolates based on their integrated phenotypic and genotypic characteristics. The reference strain ATCC12228 and RP62A are indicated by an arrow to facilitate its identification in the PCA plot.

To identify isolates with virulent-like characteristics, integrative clustering analysis was performed, including all isolates (Figure 4A). Two major groups (1 and 2) were identified, reflecting similarities in the combined genotypic and phenotypic profiles of the isolates. The reference strain RP62A, a clinically derived virulent strain was located within group 2, suggesting that this group comprises isolates with higher virulence potential. In contrast, the reference strain ATCC12228 a non-virulent strain was included in group 1, which consisted primarily of commensal or non-virulent isolates.

Regarding antibiotic resistance/susceptibility profiles, two main clusters (I and II) were identified. Cluster I comprised antibiotics to which most isolates were susceptible, and included cefoxitin, linezolid, ciprofloxacin, gatifloxacin, and ofloxacin. In contrast, Cluster II included antibiotics with higher resistance patterns among isolates, such as vancomycin, penicillin, erythromycin, and clarithromycin, among others.

In contrast, no clear grouping was observed based on the presence or absence of the analyzed genes. The distribution of resistance-associated and biofilm-related genes (*ica*D, *aap*, *mec*A, IS256, and *sar*A) did not correlate with the antimicrobial susceptibility clusters. Although some isolates in Cluster I had higher frequencies of *mec*A and biofilm-associated genes, the presence of these genes alone did not fully account for the resistance phenotype; this indicates a complex relationship between genotype and phenotypic resistance. Regarding biofilm formation, natural and trypsin-treated conditions showed heterogeneous patterns across clusters.

Because clustering analysis alone did not clearly distinguish isolates with virulent-like characteristics from the reference strains, PCA was performed to integrate the phenotypic and genotypic data from all isolates (Figure 4B).

The first two principal components (PCs) explained 37.2% of the total variance, with PC1 accounting for 21.8% and PC2 for 15.4%. Therefore, the PCA plot was interpreted as an exploratory visualization of the main patterns captured by these two components, rather than as a complete representation of the total variability within the dataset. PC1 mainly separated isolates according to their antimicrobial resistance profiles rather than traits associated with biofilm formation. In contrast, PC2 appeared to discriminate isolates based on their biofilm-forming capacity and the presence of biofilm-associated genes (Figure S2).

The PCA showed a heterogeneous distribution of isolates. While the majority clustered near the center, indicating intermediate phenotypic profiles several isolates exhibited clear separation, particularly isolates capable of producing biofilm both under natural and trypsin-treated conditions. Notably, the reference strain RP62A, included as a virulence-associated and strong biofilm-producing reference strain, showed a marked displacement from the central cluster. In contrast, the non-biofilm-producing reference strain ATCC12228 was located near the central region of the PCA plot (Figure 4B).

Using PCA as an exploratory visualization of isolate distribution, together with the biofilm-forming capacity and antimicrobial resistance/susceptibility profiles, the isolates were assigned to four groups. Isolates exhibiting moderate or strong biofilm-forming capacity together with antibiotic resistance were classified as “virulent-like.” Isolates displaying moderate or strong biofilm-forming capacity but remaining susceptible to the tested antibiotics were classified as “low virulent-like.” Isolates showing weak biofilm-forming capacity or biofilm formation only after trypsin treatment, together with antibiotic susceptibility, were classified as “commensal-like.” Finally, isolates showing no biofilm formation under either condition and antibiotic susceptibility were classified as “non-virulent-like” (Table 1). According to these predefined criteria, Bc6, Bc5, and Aax5 were classified as “virulent-like” isolates because they showed moderate biofilm-forming capacity together with resistance or intermediate susceptibility to more than half of the tested antibiotics. A second group, comprising An1, An5, Bc4, Bc7, Bn6, Bax2, and Bax8, was classified as “low virulent-like” because these isolates showed moderate biofilm-forming capacity but remained susceptible to most of the tested antibiotics. Twenty isolates were classified as “commensal-like” because they showed weak biofilm-forming capacity or biofilm formation only after trypsin treatment, together with antibiotic susceptibility. Finally, 22 isolates were classified as “non-virulent-like” because they did not show biofilm formation under either natural or trypsin-treated conditions and were antibiotic-susceptible.

Based on these analyses, isolates Bc6 and Bc5 were selected as probable virulent-like isolates, characterized by antibiotic resistance and biofilm-forming capacity. Isolate Bn5 was selected as a representative non-virulent-like isolate, as it was antibiotic-susceptible and lacked detectable biofilm formation under both natural and trypsin-treated conditions. Additionally, Bn1 was selected as a representative commensal-like isolate, since it was antibiotic-susceptible and classified as a non-biofilm producer under natural conditions but as a biofilm producer after trypsin treatment. The phenotypic and genotypic characteristics of these virulence-associated isolates are summarized in Table 2.

### 2.5. In Vitro and In Vivo Biofilm Formation Assay

**Figure 5:**
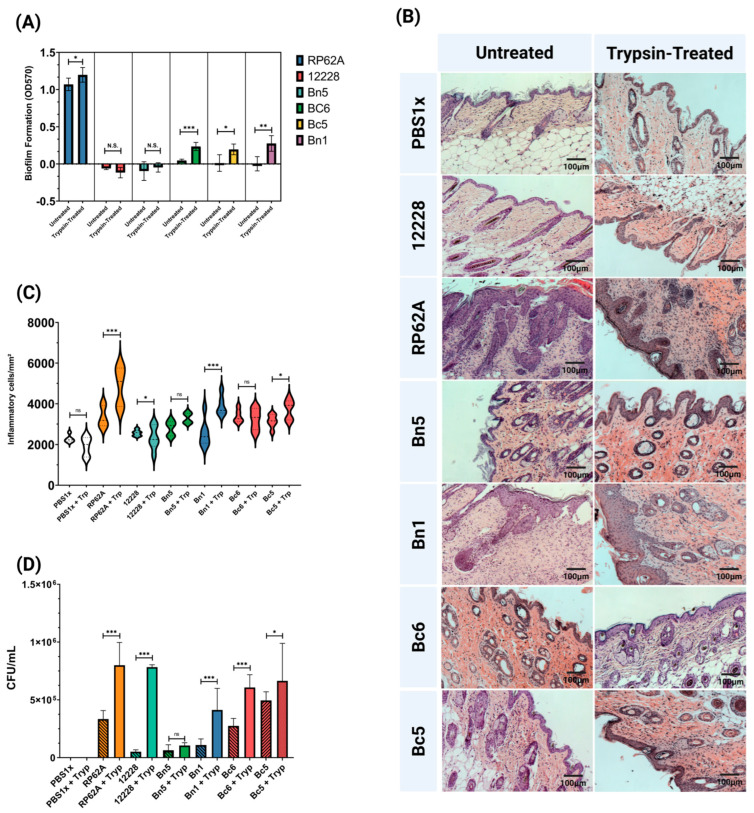
In vivo catheter colonization response induced by *Staphylococcus epidermidis* isolates. (**A**) Comparison of biofilm formation by selected isolates under natural and trypsin-treated conditions. (**B**) Representative H&E-stained sections of skin surrounding implanted catheters under untreated and trypsin-treated conditions. Scale bar = 100 µm. (**C**) Quantification of inflammatory cell infiltration (cells/mm^2^) in skin sections associated with implanted catheters under untreated vs. trypsin-treated conditions. (**D**) Viable bacterial counts (CFU/mL) recovered from explanted catheters under untreated vs. trypsin-treated conditions. For each experimental group, 18 CFU/mL values were analyzed. Statistical significance was determined using an unpaired Student’s *t*-test (* *p* < 0.05, ** *p* < 0.01, *** *p* < 0.001, ns = not significant).

Representative isolates classified according to their biofilm-forming capacity and antimicrobial susceptibility profiles were selected for in vitro and in vivo biofilm formation assays (Table 2). In vitro biofilm formation assays revealed that isolates Bc6 and Bc5 produced weak biofilm under natural conditions, which significantly increased to moderate biofilm production after trypsin treatment. The reference strain RP62A exhibited strong biofilm formation, with a significant enhancement after trypsin treatment. Notably, Bn1 showed no biofilm formation under natural conditions but acquired biofilm-forming capacity after trypsin treatment. In contrast, isolate Bn5 and the non-virulent reference strain ATCC12228 showed no biofilm formation under either condition (Figure 5A).

To determine whether the isolates possessed the capacity to colonize implanted catheters despite differences in their biofilm-forming capacity compared to the virulent strain RP62A an in vivo CCAMM was employed. Histological analysis of skin tissues from the CCAMM revealed minor differences between the experimental groups (Figure 5B). Most skin exhibited preserved epidermal architecture and normal dermal organization. However, in some cases, a slight epidermal thickening was observed, particularly in mice inoculated with the virulent reference strain RP62A and isolate Bc5. Similarly, when isolates were treated with trypsin prior to inoculation, exposed mouse skin exhibited a mild increase in epidermal thickness. Skin samples inoculated with *S. epidermidis* showed dense inflammatory cell infiltration extending into the dermis compared to phosphate-buffered saline (PBS 1×) controls. However, differences in immune cell infiltration were observed between trypsin-treated Bc5 and Bn1 isolates and natural counterparts (Figure 5C).

Upon catheter removal, viable bacterial counts were determined to quantify colonization levels in vivo (Figure 5D). The reference virulent strain RP62A exhibited the highest bacterial burden on recovered catheters. Isolates Bc6 and Bc5 showed similar levels of colonization; meanwhile, isolates Bn1 and Bn5 displayed markedly lower bacterial counts, comparable to those observed for the non-virulent reference strain ATCC12228. Following trypsin treatment, viable bacterial counts increased for RP62A, Bc6, Bc5, and Bn1, indicating the presence of trypsin-sensitive biofilm-associated *S. epidermidis* populations on the implanted catheters. In contrast, no increase in bacterial counts was observed for Bn5 following trypsin treatment.

Overall, these findings were consistent with the classification derived from the phenotypic characterization. The virulent-like isolates Bc6 and Bc5 exhibited levels of viable bacterial counts comparable to those observed for the reference strain RP62A. Although isolate Bn1 was classified as commensal-like, it exhibited increased biofilm-associated bacterial counts following trypsin treatment in both the in vitro and in vivo models. In contrast, isolate Bn5 classified as non-virulent-like, consistently displayed low levels of colonization and no evidence of trypsin-sensitive biofilm formation under either experimental condition.

### 2.6. In Vivo Competition and Colonization in a Catheter-Associated Model

Multiple bacterial species coexist within the same anatomical skin site. In this environment, strains with different phenotypic characteristics, including those with or without the capacity to adhere to and colonize catheter surfaces may contaminate a medical device. Based on this concept, a competitive assay for device-associated biofilm formation on a catheter was performed between the virulent-like isolate Bc5 and the non-virulent isolate Bn5. Both isolates were simultaneously inoculated into the catheter at the same bacterial load.

**Figure 6:**
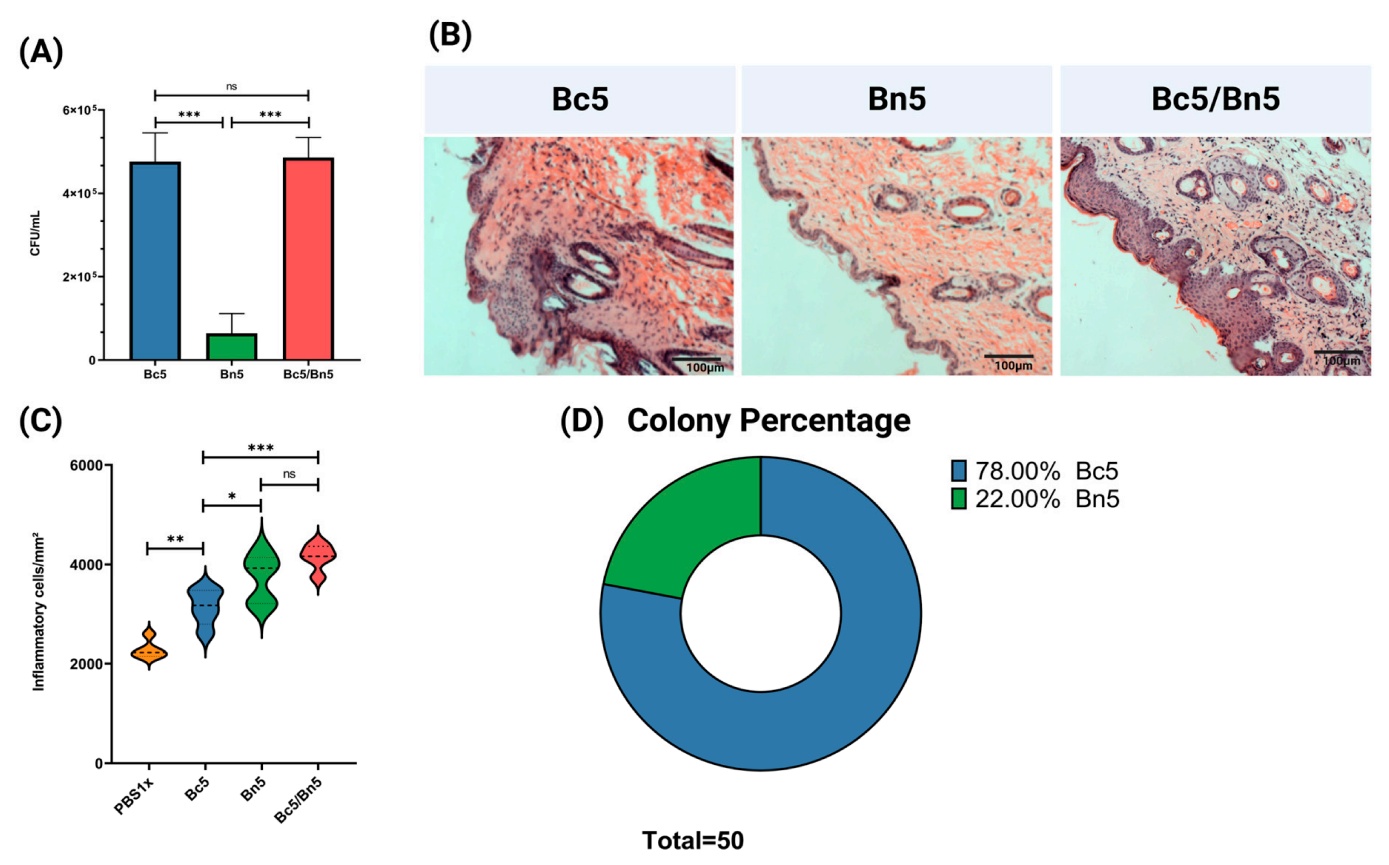
In vivo competition assay in a catheter-associated contamination model. (**A**) Viable bacterial counts (CFU/mL) recovered from explanted catheters inoculated with *S. epidermidis* isolates Bc5, Bn5, or a combined inoculum (Bc5/Bn5). For each experimental group, 18 CFU/mL values were analyzed. (**B**) Representative H&E-stained sections of skin surrounding implanted catheters. (**C**) Quantification of inflammatory cell infiltration (cells/mm^2^) in skin sections associated with implanted catheters. Scale bar = 100 µm (**D**) Colony distribution analysis of 50 recovered CFUs from the combined inoculum. Statistical significance was determined using one-way ANOVA followed by Tukey’s multiple comparison test (* *p* < 0.05, ** *p* < 0.01, *** *p* < 0.001, ns = not significant).

The number of bacteria recovered (CFU/mL) from the catheter inoculated with the Bc5/Bn5 combination was very similar to that recovered when the catheter was inoculated with Bc5 alone (Figure 6A), indicating no increase in biofilm biomass. Histological analysis of mouse skin from the Bc5/Bn5 combination was similar to that of skin contaminated with the Bc5 isolate alone (Figure 6B); the same was observed for the level of cellular infiltration in the dermis (Figure 6C).

Genotypic analysis of the bacteria recovered from the Bc5/Bn5 combination showed that 78% of the bacteria corresponded to the Bc5 genotype (*aap*^+^/*ica*D^−^ genotype), with the remaining proportion corresponding to Bn5 (*aap*^−^/*ica*D^+^ genotype; Figure 6D). Altogether, these results indicate that isolate Bc5 showed a competitive advantage over isolate Bn5 in catheter colonization when both isolates coexisted within the same environment.

## 3. Discussion

In recent years, *S. epidermidis* has become widely recognized as an opportunistic pathogen capable of causing human infections, particularly through the contamination of medical devices [31]. Despite strict hygiene protocols and preventive measures during device handling in hospital settings, cases of contamination and infection caused by *S. epidermidis* continue to occur. Suggested sources of contamination include the hospital environment, the skin of healthcare workers, or the skin of patients themselves [27]. This study identified the presence of *S. epidermidis* isolates with virulence-like characteristics similar to those of the virulent clinical reference strain RP62A on the skin of dermatologically healthy individuals with no history of hospitalization during the three years prior to sampling. However, the combined occurrence of virulent-like and low virulent-like isolates was relatively low (19.23%) within a small collection of isolates (*n* = 52). Although the number of isolates analyzed was limited, the presence of these virulent-like isolates among dermatologically healthy individuals supports the possibility that the skin microbiota may serve as a reservoir of *S. epidermidis* that may contribute to medical device contamination during invasive medical procedures, including device implantation. 

In the context of nosocomial infection by *S. epidermidis*, several studies have reported that contamination may originate from healthcare workers [31]. One particular study found staphylococcal infection in neonates to be partly attributable to both the mother and hospital staff; while mothers represented the main source of neonatal infection associated with *Staphylococcus haemolyticus*, healthcare workers were the main carriers of *S. epidermidis* to neonates and young infants at the Ho Teaching Hospital in Ghana [32]. Similarly, a study carried out in Greece at a tertiary-care hospital demonstrated that a single clone of *S. epidermidis* colonized 40% of the clinical staff and was linked to patient infections [33]. Other studies have reported that 75% of hospital staff have methicillin-resistant *S. epidermidis* on their skin and nasal cavities, with nurses showing a higher prevalence of methicillin-resistant *S. epidermidis* compared to other medical staff; this further suggests that the skin of healthcare staff may serve as a vehicle for infection [29]. Meanwhile, inanimate objects, like phones or stethoscopes used by healthcare professionals, should also be regarded as potential sources of contamination. In a hospital in Ethiopia, a study revealed that 60.1% of 375 inanimate objects collected from 191 participants were contaminated with microorganisms, with *S. aureus* being the most frequently isolated (22.1%), followed by *S. epidermidis* (17.0%) [34]. These findings support the idea that *S. epidermidis* associated with human skin may contribute to bacterial transmission to newborn skin and to medical devices. In this context, our study adds further evidence by showing that dermatologically healthy individuals with no association with hospital staff may also harbor *S. epidermidis* isolates with the capacity to contaminate a catheter in different anatomical skin regions.

The most important virulence factors in *S. epidermidis* are biofilm production and antibiotic resistance. Biofilm production is more pronounced in *S. epidermidis* isolates recovered from infected medical devices than in strains isolated from the skin of dermatologically healthy subjects, supporting the role of this bacterium as a potential pathogen [26,27,28]. However, commensal *S. epidermidis* isolates from healthy skin collected from multiple individuals may also be capable of inducing biofilm production when treated with an exogenous protease, such as trypsin [35]. In a population of biofilm-negative in vitro *S. epidermidis* isolates from healthy skin, it was demonstrated that treatment with trypsin increased the number of biofilm-producing isolates from 17% up to 79%, producing functional biofilm [35]. This indicates that skin-dwelling *S. epidermidis* has the capacity to produce biofilm under proteolytic conditions. Trypsin cleaves the beta subunit of the Aap protein, enabling it to bind to another proteolytically processed Aap molecule present in other bacteria, ultimately forming a proteinaceous biofilm [20,35]. In our study, trypsin treatment increased the number of biofilm-producing isolates recovered from healthy individuals, with these isolates showing a high frequency (70%) of the *aap* gene. These observations suggest that host proteases could induce biofilm production during the implantation of medical devices contaminated with *S. epidermidis* originating from the patient’s own skin. This hypothesis is supported by evidence showing that the presence of neutrophils or neutrophil-derived proteases such as cathepsin G, cathepsin B, proteinase-3, and metalloproteinase-9 can induce biofilm formation in a biofilm-negative *S. epidermidis* strain from healthy skin [36]. This would explain our in vivo results, where both virulent-like (Bc5, Bc6) and commensal-like (Bn1) isolates treated with trypsin showed increased biofilm formation on the CCAMM, compared with their untreated counterparts.

Competition between isolates within a given anatomical skin site to contaminate a medical device and form biofilm depends on the capabilities of the respective bacteria. Our data suggest that the virulent-like isolate Bc5 was more competitive than the non-virulent-like isolate Bn5 during device-associated biofilm formation on implanted catheters. This supports the possibility that *S. epidermidis* isolates with virulence-associated traits may have a greater capacity to contaminate catheter surfaces and establish biofilms. In addition, previous studies have suggested that competition can occur within biofilm communities, including mixed biofilms composed of different microbial species, such as *Candida albicans* and *S. epidermidis* [37]. Likewise, competition among distinct *S. epidermidis* strains may influence biofilm composition, as Agr type III strains have been shown to outcompete and predominate over Agr type I and II strains under certain environmental conditions [38].

The most important finding of this study was the identification of *S. epidermidis* isolates recovered from healthy skin that were able to colonize implanted catheters and form device-associated biofilms at levels comparable to those observed for the virulent reference strain RP62A in an in vivo model, supporting the idea that healthy skin may harbor *S. epidermidis* with virulence-associated traits. Although the combined frequency of virulent-like and low virulent-like isolates identified in this study was relatively low (19.23%), these findings should be interpreted in the context of the clinical relevance of the catheter contamination. Epidemiological data from the United States indicate that the catheter contamination occur in approximately 4–5 cases per 1000 intravenous catheter insertions in intensive care settings, with *S. epidermidis* accounting for nearly 22% of these infections [39]. The low frequency of *S. epidermidis* isolates with catheter-colonizing potential suggests that dermatologically healthy individuals may harbor only a limited proportion of skin-associated isolates capable of contaminating medical devices. In the present study, virulent-like and low virulent-like isolates were detected only in subjects A and B, whereas the remaining subjects mainly harbored commensal-like or non-virulent-like isolates. This observation suggests that the presence of *S. epidermidis* isolates with virulence-associated traits is not uniformly distributed among healthy individuals. Such variability may be influenced by host- and environment-related factors, including sex, geographic origin, environmental exposure, hygiene habits, cosmetic use, and other variables that can shape the composition and phenotypic diversity of *S. epidermidis* populations on healthy skin.

In a previous study, Zhou et al. [30] analyzed 1500 *S. epidermidis* isolates recovered from five healthy individuals across 12 anatomical skin sites. Whole-genome sequencing was used to characterize the composition and dynamics of *S. epidermidis* populations on healthy skin. Their findings revealed substantial genetic diversity among *S. epidermidis* populations and showed that different founder strains coexisted within each individual and at distinct anatomical skin sites, further highlighting the diversity among isolates recovered from healthy skin. In this regard, our results were very similar, showing high phenotypic and genotypic diversity between individuals. Although this study provides evidence that dermatologically healthy individuals may harbor *S. epidermidis* isolates with the capacity to contaminate medical devices, several limitations should be acknowledged. The relatively small number of isolates analyzed (*n* = 52), which were obtained from only five healthy individuals within a narrow age range and from a single geographic region. Moreover, because strain genotyping or whole-genome sequencing was not performed, the possibility that some isolates recovered from the same subject or anatomical site may be closely related cannot be excluded. In addition, the isolate classification should be interpreted as a phenotypic categorization supported by exploratory PCA visualization, since group assignment was based on biofilm-forming capacity and antimicrobial resistance/susceptibility profiles. Another limitation is that only a small number of representative isolates was evaluated in vivo; therefore, future studies should include a larger number of isolates from each group to validate their catheter-colonizing capacity. Finally, although CCAMM is useful for evaluating catheter colonization and local biofilm-associated responses, it does not fully reproduce the complexity of human device-related infections. Therefore, future studies should include larger collections of *S. epidermidis* isolates recovered from individuals from different geographic regions, together with strain typing, deeper genomic characterization, and broader in vivo validation to better define the frequency, diversity, and clinical relevance of virulence-associated traits within skin-associated *S. epidermidis* populations. Overall, our findings suggest that patient-derived skin microbiota may represent a potential source of *S. epidermidis* strains capable of contaminating medical devices.

## 4. Materials and Methods

### 4.1. Isolation of S. epidermidis from Different Anatomical Skin Sites of Healthy Subjects

*S. epidermidis* isolates were obtained from the skin of five dermatologically healthy subjects through swab sampling. Samples were collected from four anatomical sites: the head, arms, armpits, and nose. Healthy subjects were defined as individuals aged 25–30 years, without visible dermatological disease, active infection, or hospitalization within the previous three years prior to sampling. In addition, participants confirmed that they were not associated with hospitals or healthcare environments and had no direct contact with hospitalized patients.

Swab samples were streaked onto mannitol salt agar plates (Becton Dickinson, Franklin Lakes, NJ, USA) and incubated at 37 °C for 24 h. Colonies that did not ferment mannitol, indicated by the absence of a yellow halo, and showed Gram-positive cocci arranged in clusters were selected. Mannitol-negative colonies were further evaluated by coagulase testing and novobiocin susceptibility. Coagulase-negative and novobiocin-susceptible isolates were presumptively identified as *S. epidermidis*. Species confirmation was performed by PCR amplification of the 16S rRNA *S. epidermidis*-specific primers as previously described by Juárez-Verdayes et al. 2006 [40]. A total of 52 confirmed *S. epidermidis* isolates were included in the study. All confirmed isolates were preserved in tryptic soy broth (TSB) supplemented with 20% glycerol and stored at −80 °C until subsequent phenotypic and genotypic characterization studies. The clinical *S. epidermidis* reference strain RP62A (ATCC 35984), commercially obtained from ATCC (Manassas, VA, USA) and originally isolated from a case of catheter sepsis in Tennessee, USA, was used as a biofilm-producing clinical reference strain. *S. epidermidis* ATCC 12228, also commercially obtained from ATCC, was used as a non-biofilm-producing reference strain.

The study was conducted in accordance with the Declaration of Helsinki, and the protocol was approved by the Ethics Committee of the “Escuela Nacional de Ciencias Biológicas, Instituto Politécnico Nacional”, Mexico City, Mexico (Approval No. HUM-001-2021) in June 2021. The isolates were obtained in Mexico City, Mexico. Informed consent was obtained from all participants prior to their inclusion in the study.

### 4.2. Genotypic Characterization

Genomic DNA was extracted from all isolates as previously described [41]. The presence of biofilm-associated genes: *ica*A and *ica*D, which are components of the icaADBC operon and participate in the synthesis of the exopolysaccharide PIA/PNAG; *aap*, which encodes the cell wall-anchored accumulation-associated protein involved in protein-dependent biofilm formation and protease-mediated biofilm induction; and *sar*A, which encodes a transcriptional regulator associated with biofilm formation and icaADBC operon regulation. The insertion sequence IS256, a mobile genetic element that may disrupt or modulate genes involved in biofilm formation, was also evaluated. Finally, the presence of *mec*A, which encodes penicillin-binding protein 2a and is associated with methicillin resistance. The presence of these genes and the insertion sequence IS256 was determined by polymerase chain reaction (PCR) using Taq DNA polymerase (Bioline, Singapore) following the manufacturer’s instructions. Primer sequences are listed in Table A1.

The PCR conditions were as follows: initial denaturation at 95 °C for 1 min, followed by 30 cycles of denaturation at 95 °C for 40 s, annealing at 55 °C for 30 s, and extension at 72 °C for 40 s. PCR products were analyzed by electrophoresis on 1% agarose gels stained with ethidium bromide.

### 4.3. In Vitro Biofilm Formation

In vitro biofilm formation was assessed by initially cultivating a single bacterial colony overnight in TSB. The cultures were subsequently diluted at a ratio of 1:200 in fresh TSB, and the optical density was measured at 600 nm (OD_600_). These diluted cultures were then inoculated into the individual wells of 96-well flat-bottom microtiter plates (Nunc) and incubated at 37 °C for 24 h. After incubation, the wells were washed with PBS 1× and stained with 0.1% (*w*/*v*) crystal violet. Adherent biofilm biomass was quantified by measuring the optical density at 570 nm (OD_570_) using a Multiskan GO microplate spectrophotometer (Thermo Fisher Scientific, Waltham, MA, USA). All assays were performed in six independent replicates, and the mean OD values were reported (Table S1).

For trypsin-induced biofilm formation, the same procedure was followed, except that the cultures were diluted in TSB supplemented with trypsin at a final concentration of 0.2 µg/mL.

Biofilm-producing and non-biofilm-producing isolates were differentiated using the microtiter plate assay described by Christensen et al., 1985 [42], based on crystal violet staining and OD_570_ measurement. The OD cut-off value (ODc) was calculated using the following formula:

ODc = mean OD_570_ of the negative control + 3(SD of the negative control)

In this study, the calculated ODc was 0.0367. Therefore, isolates with OD_570_ values ≤ 0.0367 were considered non-biofilm producers. Isolates with OD_570_ values > 0.0367 and ≤0.120 were classified as weak biofilm producers, isolates with OD_570_ values > 0.120 and ≤0.240 were classified as moderate biofilm producers, and isolates with OD_570_ values > 0.240 were classified as strong biofilm producers.

### 4.4. Antimicrobial Susceptibility Test

The antimicrobial susceptibility of the bacterial isolate was assessed using the Kirby–Bauer disk diffusion method. A single bacterial colony was grown overnight on Mueller–Hinton agar at 37 °C and subsequently inoculated into fresh Mueller–Hinton broth, until the turbidity reached 0.5 at OD_600_. The standardized bacterial suspension was evenly distributed onto Mueller–Hinton agar plates, followed by the application of antibiotic disks containing penicillin, erythromycin, vancomycin, clarithromycin, linezolid, co-trimoxazole, ciprofloxacin, gatifloxacin, azithromycin, ofloxacin, clindamycin, or cefoxitin. The plates were incubated at 37 °C for 24 h. Subsequently, the diameters of the inhibition zones were measured.

### 4.5. Murine Catheter-Associated Contamination Model

Female BALB/c mice, 6–8 months of age, were obtained from the animal facility of the “Escuela Nacional de Ciencias Biológicas, Instituto Politécnico Nacional”, and used for the catheter contamination assay in a mouse back model (CCAMM) [43]. Before inclusion in the study, animals were clinically inspected by a veterinarian, and only mice without visible alterations in the skin, eyes, or ears were included. The minimum number of mice required for the experimental design was determined using G*Power software version 3.1.9.7 and in accordance with the bioethical recommendations of the Instituto Politécnico Nacional. Sample size estimation was performed using a significance level of α = 0.05 and a statistical power of 1 − β = 0.80. The catheter contamination assay was performed in a blinded manner. Mice were randomly assigned to experimental groups according to the inoculated isolate or reference strain and treatment condition. Each experimental condition included six animals. Each isolate or reference strain was evaluated under untreated and trypsin-treated conditions, with a total of 72 mice assigned to the bacterial inoculation groups. An additional group of six mice inoculated with PBS 1× was included as the negative contamination control, resulting in a total of 78 mice used in the catheter contamination assay.

Mice were anesthetized by intraperitoneal injection of pentobarbital (50 mg/g body weight). A small incision was made on the dorsal surface, and a 1 cm segment of a sterile 14-gauge catheter was inserted subcutaneously. The incision was then surgically closed. An inoculum of approximately 1.5 × 10^8^ CFU in 20 µL sterile PBS was injected into the catheter lumen. For trypsin-treated groups, the same bacterial inoculum was supplemented with trypsin (0.2 µg/mL) and injected into the implanted catheters. Fourteen days post-inoculation, the mice were euthanized. The catheters were carefully removed for bacterial recovery, and skin biopsies surrounding the catheter implantation site were collected for histological analysis.

Catheters were sonicated at 200 Hz for 5 min twice to detach adherent bacteria. Bacteria were homogenized, serially diluted, and plated on mannitol salt agar. Each dilution was plated in three technical replicates. The plates were incubated at 37 °C for 24 h. For CFU quantification, plates containing 30–300 colonies were selected, and colony-forming units per milliliter (CFU/mL) were calculated for each technical replicate using the following formula:

CFU/mL = (number of colonies × dilution factor)/volume plated (mL)

All animal experiments were carried out following the National Institutes of Health guidelines for the care and use of laboratory animals. The experimental protocol was reviewed and approved by the internal Ethics Committee of the “Escuela Nacional de Ciencias Biológicas, Instituto Politécnico Nacional”, Mexico City, Mexico (ZOO-001-2021).

### 4.6. In Vivo Competition Assay

For the in vivo competition assay, mice were handled following the same procedures described in Section 4.5. Three CCAMM experimental groups were designed, with six mice included in each group. One group was inoculated with the virulent-like isolate Bc5, another group was inoculated with the non-virulent-like isolate Bn5, and a third group was inoculated with a mixed bacterial suspension containing both Bc5 and Bn5. El isolate Bc5 was mixed with isolate Bn5 at equal bacterial amounts for both isolates (approximately 1.5 × 10^8^ CFU in a total volume of 20 µL). The mixture (20 µL) was inoculated into a catheter inserted in the dorsal region of the mouse, following the procedure described in Section 4.5. At the end of the incubation period, CFU/mL were determined by plating on mannitol salt agar in three technical replicates. Subsequently, 50 colonies were randomly selected from mannitol salt agar plate obtained from the viable count. Genomic DNA was extracted from the selected bacterial colonies, and PCR was performed to determine the presence of the *aap* and *ica*D genes in each isolate, following the procedure described in Section 4.2. Identification of the Bc5 isolate among the recovered colonies was based on the *aap*^+^/*ica*D^−^ genotype; the Bn5 isolate was identified by the *aap*^−^/*ica*D^+^ genotype. The frequency of each genotype among the recovered bacteria was then determined.

### 4.7. Inflammatory Cell Image Analysis

Skin biopsies surrounding the catheter implantation site were collected for histological analysis. For each isolate and experimental condition, two independent experimental repeats were performed, with three animals per repeat, resulting in six animals per group. Biopsies were paraffin-embedded, and five serial sections were obtained from each biopsy for hematoxylin and eosin (H&E) staining, resulting in 30 H&E-stained sections per isolate and condition.

Using an inverted microscope (Zeiss Axiovert 5 TL, Carl Zeiss Microscopy GmbH, Jena, Germany), four representative images were acquired from each H&E-stained section at 20× magnification, resulting in 120 images analyzed per isolate and condition. Image analysis was performed using QuPath version 0.7.0 with the Cellpose-SAM extension to assist with automated segmentation and quantification of inflammatory infiltrating cells.

### 4.8. Data Analysis

PCA and heatmap analyses were performed by integrating phenotypic (antibiotic susceptibility, biofilm formation under natural and trypsin-treated conditions) and genotypic (presence/absence of *ica*D, *aap*, *mec*A, IS256, and *sar*A) data from *S. epidermidis* isolates using RStudio (version 4.5.1). Prior to PCA, variables were scaled and centered to ensure comparability. Principal components were selected based on the proportion of variance explained and inspection of scree plots, retaining components that cumulatively explained the variance. Heatmap visualization and hierarchical clustering were performed using Euclidean distance and complete linkage, clustering variables (antibiotics and genes) based on their similarity patterns across isolates.

For in vivo and in vitro experiments, statistical analyses were conducted using GraphPad Prism 9. Data are presented as mean ± SEM. Normality was assessed for each dataset using the Shapiro–Wilk test, and equality of variances was evaluated using the Brown–Forsythe test. For pairwise comparisons, Student’s *t*-tests were applied, as appropriate. For comparisons among more than two groups, one-way ANOVA followed by Tukey’s multiple comparison test was used. A *p*-value < 0.05 was considered statistically significant. For CFU/mL quantification, the bacterial suspension recovered from each catheter was plated in technical triplicate. Accordingly, 18 technical CFU/mL measurements were obtained for each experimental group.

## 5. Conclusions

In conclusion, our data suggest that a small group of young dermatologically healthy individuals from Mexico City, with no reported contact with hospital environments, may harbor phenotypically heterogeneous *S. epidermidis* isolates across different anatomical skin sites. Among these isolates, some displayed virulence-associated characteristics similar to those of the reference strain RP62A, including enhanced biofilm-forming capacity in vitro and the ability to colonize implanted catheters in vivo. These findings highlight the potential role of healthy skin as a reservoir of *S. epidermidis* isolates capable of contaminating medical devices. Moreover, this study provides a basis for future investigations involving larger isolated collections, broader geographic sampling, and deeper genomic characterization to determine the frequency, diversity, and clinical relevance of skin-associated *S. epidermidis* populations.

## Figures and Tables

**Table 1:** Classification of *S. epidermidis* isolates based on biofilm-forming capacity and antimicrobial susceptibility profiles.

	Subject A				Subject B				Subject C				Subject D				Subject E			
	Head	Nose	Arm	Arm Pit	Head	Nose	Arm	Arm Pit	Head	Nose	Arm	Arm Pit	Head	Nose	Arm	Arm Pit	Head	Nose	Arm	Arm Pit
Moderate/strong biofilm-producing, antibiotic-resistant virulent-like isolates			Aax5		Bc5 Bc6															
Moderate/strong biofilm-producing, antibiotic-susceptible low virulent-like isolates		An1 An5			Bc4	Bn6		Bax2 Bax8	Bc7											
Weak biofilm-producing, antibiotic-susceptible commensal-like isolates	Ac1 Ac8a Ac8b				Bc2 Bc9	Bn1 Bn2 Bn3 Bn9	Bax9		Bc8 Cc9	Bn4 Bn7 Bn8	Cax7 Cax9		Bc10 Dc10					En8		
Non-biofilm-producing, antibiotic-susceptible non-virulent-like isolates		An6 Bax10	Ab5 Ab7		Bc1	Bn5	Bax7		Cc10	Cn4 Cn5	Cax4		Bc3 Dc9	Bn10		Dax4	Ec3 Ec9 Ec10	En1 En7 En9 En10		

**Table 2:** Phenotypic and genotypic characteristics of selected *S. epidermidis* isolates used in the catheter contamination assay in a mouse back model (CCAMM).

Isolate	Biofilm (Natural)	Biofilm (Trypsin-Treated)	Cef	Lin	Cip	Gat	Oflo	Clin	Azi	Ery	Cla	CoT	Pen	Van	*ica*D	*aap*	*ica*A	*mec*A	IS256	*sar*A
RP62A	+	+	S	S	S	S	S	S	I	I	I	I	I	I	+	+	+	+	+	+
Bc6	+	+	S	R	R	S	S	S	R	R	R	I	I	I	-	+	-	-	-	-
Bc5	+	+	S	S	S	S	S	S	I	I	I	I	I	I	-	+	-	-	-	-
Bn1	-	+	S	S	S	S	S	I	R	I	I	R	I	I	-	+	-	-	-	-
ATCC12228	-	-	S	S	S	S	S	R	R	R	R	R	I	I	-	+	-	-	+	+
Bn5	-	-	S	S	S	S	S	S	R	R	I	R	R	I	+	-	-	-	-	-

Abbreviations: Cef = cefoxitin; Lin = linezolid; Cip = ciprofloxacin; Gat = gatifloxacin; Oflo = ofloxacin; Clin = clindamycin; Azi = azithromycin; Ery = erythromycin; Cla = clarithromycin; CoT = co-trimoxazole; Pen = penicillin; Van = vancomycin; S = susceptible; I = intermediate; R = resistant.

## Data Availability

The original contributions presented in this study are included in the article. Further inquiries can be directed to the corresponding author.

## Supplementary Materials

The following supporting information can be downloaded at: https://www.mdpi.com/article/10.3390/ijms27156611/s1.

## Abbreviations

The following abbreviations are used in this manuscript:

CCAMM	catheter contamination assay in a mouse back model
PCA	Principal component analysis
PBS 1×	phosphate-buffered saline

## Appendix A

**Table A1:** Primer sequences.

Primer	Sequence
*Aap* Forward	5′-CATTGACATACACTCCTAAGC-3′
*Aap* Reverse	5′-CCAAATATGAACAATGATCCG-3′
*IcaA* Forward	5′- TCTCTTGCAGGAGCAATCAA-3′
*IcaA* Reverse	5′- AGGCACTAACATCCAGCA-3′
*IcaD* Forward	5′-ATGGTCAAGCCCAGACAGAG-3′
*IcaD* Reverse	5′-CGTGTTTTCAACATTTAATGCA-3′
*SarA* Forward	5′-TCTTTCATCGTGTTCATTACG-3′
*SarA* Reverse	5′-AATCAGCTTTGAAGAATTTGC-3′
*IS256* Forward	5′-TGAAAAGCGAAGAGATTCAAAGC-3′
*IS256* Reverse	5′-ATGTAGGTCCATAAGAACGGC-3′
